# Cluster analysis to estimate the risk of preeclampsia in the high-risk Prediction and Prevention of Preeclampsia and Intrauterine Growth Restriction (PREDO) study

**DOI:** 10.1371/journal.pone.0174399

**Published:** 2017-03-28

**Authors:** Pia M. Villa, Pekka Marttinen, Jussi Gillberg, A. Inkeri Lokki, Kerttu Majander, Maija-Riitta Ordén, Pekka Taipale, Anukatriina Pesonen, Katri Räikkönen, Esa Hämäläinen, Eero Kajantie, Hannele Laivuori

**Affiliations:** 1 Obstetrics and Gynaecology, University of Helsinki and Helsinki University Hospital, Helsinki, Finland; 2 Helsinki Institute for Information Technology HIIT, Department of Computer Science, Aalto University, Espoo, Finland; 3 Immunobiology, Research Programs Unit, University of Helsinki, Helsinki, Finland; 4 Medical and Clinical Genetics, University of Helsinki and Helsinki University Hospital, Helsinki, Finland; 5 Bacteriology and Immunology, University of Helsinki, Helsinki, Finland; 6 Institute for Archaeological Sciences, University of Tübingen, Tübingen, Germany; 7 Obstetrics and Gynecology, Kuopio University Hospital, Kuopio, Finland; 8 Suomen Terveystalo Oy, Kuopio, Finland; 9 Department of Psychology and Logopedics, University of Helsinki, Helsinki, Finland; 10 HUSLAB, University of Helsinki and Helsinki University Hospital, Helsinki, Finland; 11 National Institute for Health and Welfare, Helsinki and Oulu, Finland; 12 PEDEGO Research Unit, MRC Oulu, Oulu University Hospital and University of Oulu, Oulu, Finland; 13 Children’s Hospital, Helsinki University Hospital and University of Helsinki, Helsinki, Finland; 14 Institute for Molecular Medicine Finland, University of Helsinki, Helsinki, Finland; Hungarian Academy of Sciences, HUNGARY

## Abstract

**Objectives:**

Preeclampsia is divided into early-onset (delivery before 34 weeks of gestation) and late-onset (delivery at or after 34 weeks) subtypes, which may rise from different etiopathogenic backgrounds. Early-onset disease is associated with placental dysfunction. Late-onset disease develops predominantly due to metabolic disturbances, obesity, diabetes, lipid dysfunction, and inflammation, which affect endothelial function. Our aim was to use cluster analysis to investigate clinical factors predicting the onset and severity of preeclampsia in a cohort of women with known clinical risk factors.

**Methods:**

We recruited 903 pregnant women with risk factors for preeclampsia at gestational weeks 12^+0^–13^+6^. Each individual outcome diagnosis was independently verified from medical records. We applied a Bayesian clustering algorithm to classify the study participants to clusters based on their particular risk factor combination. For each cluster, we computed the risk ratio of each disease outcome, relative to the risk in the general population.

**Results:**

The risk of preeclampsia increased exponentially with respect to the number of risk factors. Our analysis revealed 25 number of clusters. Preeclampsia in a previous pregnancy (n = 138) increased the risk of preeclampsia 8.1 fold (95% confidence interval (CI) 5.7–11.2) compared to a general population of pregnant women. Having a small for gestational age infant (n = 57) in a previous pregnancy increased the risk of early-onset preeclampsia 17.5 fold (95%CI 2.1–60.5). Cluster of those two risk factors together (n = 21) increased the risk of severe preeclampsia to 23.8-fold (95%CI 5.1–60.6), intermediate onset (delivery between 34^+0^–36^+6^ weeks of gestation) to 25.1-fold (95%CI 3.1–79.9) and preterm preeclampsia (delivery before 37^+0^ weeks of gestation) to 16.4-fold (95%CI 2.0–52.4). Body mass index over 30 kg/m^2^ (n = 228) as a sole risk factor increased the risk of preeclampsia to 2.1-fold (95%CI 1.1–3.6). Together with preeclampsia in an earlier pregnancy the risk increased to 11.4 (95%CI 4.5–20.9). Chronic hypertension (n = 60) increased the risk of preeclampsia 5.3-fold (95%CI 2.4–9.8), of severe preeclampsia 22.2-fold (95%CI 9.9–41.0), and risk of early-onset preeclampsia 16.7-fold (95%CI 2.0–57.6). If a woman had chronic hypertension combined with obesity, gestational diabetes and earlier preeclampsia, the risk of term preeclampsia increased 4.8-fold (95%CI 0.1–21.7). Women with type 1 diabetes mellitus had a high risk of all subgroups of preeclampsia.

**Conclusion:**

The risk of preeclampsia increases exponentially with respect to the number of risk factors. Early-onset preeclampsia and severe preeclampsia have different risk profile from term preeclampsia.

## Introduction

Preeclampsia affects 3%[1] of all pregnancies. It is a systemic disease with a multifactorial background. Preeclampsia is diagnosed when a pregnant woman develops hypertension and proteinuria after 20 weeks of gestation. Recent recommendation by the American College of Obstetricians and Gynecologists [2] further proposes that in the absence of proteinuria, preeclampsia could be diagnosed when newly diagnosed hypertension occurs in association with thrombocytopenia, impaired liver function, new development of renal insufficiency, pulmonary edema, or new-onset cerebral or visual disturbances.

Preeclampsia can be life-threatening both to the mother and the unborn child. Due to its multifactorial etiology, the outcome and progression of preeclampsia is challenging to predict. The role of the placenta is important in the pathogenesis, and the clinical findings are a consequence of endothelial dysfunction [3]. The disease can be roughly divided into early-onset and late-onset subtypes which evidently rise from different etiopathogenic backgrounds [4]. Early-onset disease is associated with placental dysfunction. It is often accompanied by intrauterine growth restriction, the risk runs in families and women with history of early-onset preeclampsia have an increased risk of cardiovascular disease later in life [5]. Late-onset preeclampsia develops due to metabolic disturbances, obesity, diabetes, lipid dysfunction, and inflammation [6] all of which affect endothelial function. Placental hypoplasia and vascular lesions frequently present in early-onset disease are often absent in the late-onset disease [7].

Using vasoactive markers, placental growth factor (PlGF) and soluble vasoactive endothelial growth factor reseptor-1 (s-Flt1), preeclampsia can be divided into angiogenic and non-angiogenetic subgroups [8]. Early-onset and severe preeclampsia seem to form predominantly the angiogenetic subtype and non-angiogenic subtype has milder and later course of the disease [9].

Recently encouraging progress has been achieved in the field of preeclampsia prevention [10]. Meta-analyses show that low-dose aspirin initiated in the early precnancy in high-risk women may prevent early and severe preeclampsia or at least delay the onset of the disease [11,12]. However, preeclampsia is a heterogenic disorder. There are great challenges in predicting who will eventually develop severe or early-onset disease and, accordingly, benefit most from the use of aspirin.

The present study explores the features of this high-risk cohort.

The aim was to explore predicting factors affecting the onset and severity of preeclampsia. We used cluster analysis in a prospectively collected cohort of women with known clinical risk factors for preeclampsia. We will discuss the findings in the context of current recommendations for initiating aspirin to prevent preeclampsia in high-risk women.

## Methods

### The Predo Project

The study cohort consists of women recruited in the multidisciplinary Prediction and Prevention of Preeclampsia and Intrauterine Growth Restriction (PREDO) Project between September 2005 and December 2009 [13,14]. The project has three arms: obstetric [15](including an aspirin trial [13]), genetic [16], and psychological [17]. We recruited 972 pregnant women with risk factors for preeclampsia and 110 randomly selected pregnant women without known risk factors as a comparison group at 12^+0^ to 13^+6^ weeks and days of gestation. The recruitment took place when the women attended the first ultrasound screening in one of the ten hospital maternity clinics participating in the PREDO Project; Women´s Hospital, Kätilöopisto Maternity hospital and Jorvi Hospital at Helsinki University Central Hospital, Hyvinkää Hospital, Kanta-Häme Central Hospital, Päijät-Häme Central Hospital, Tampere University Hospital, Kuopio University Hospital, Northern Karelia Central Hospital, and Iisalmi Hospital. The study protocol has been approved by the Ethics Committee of the Helsinki and Uusimaa Hospital District and by the participating hospitals. A written informed consent was obtained from all participants.

#### Inclusion criteria and definitions

The inclusion and exclusion criteria for the PREDO Project are presented in Table 1. Women with one or more of the risk factors for preeclampsia were invited to participate in the order of arrival unless any of the exclusion criteria were present. We performed uterine artery blood flow measurements by Doppler ultrasound for all participants at 12^+0^ to 13^+6^ weeks of gestation. Women who had bilateral second-degree notch were allocated to the medication group. These women (n = 152) were randomized to receive aspirin in low dose (100 mg/day) or placebo until 35^+0^ weeks of gestation, or delivery. Women with risk factors but no aforementioned ultrasound finding were allocated in the follow-up groups. We also recruited 110 women without known risk factors as a control group. All study participants filled a questionnaire concerning their health. Pregnancy data were collected from the medical records of maternity clinics and hospitals.

**Table 1:** Number of women included in the cluster analysis with each risk factor. A participant may have one or more risk factors.

Risk factor	N	%
Pre-eclampsia in a previous pregnancy	222	4.6%
Small for gestational age infant in a previous pregnancy	106	11.7%
Chronic hypertension	136	15.1%
Gestational diabetes, diet-treated	90	10%
Gestational diabetes, insulin treated	12	1.3%
Body mass index over 30 kg/m^2^	353	39.1%
Age under 20 years	27	3%
Age over 40 years	149	16.5%
Systemic lupus erythematosus	4	0.4%
Sjögren’s syndrome	13	1.4%
Type 1 diabetes	18	2.0%
Previous fetal demise	37	4.1%

### Participants in the cluster analysis

Participants with risk factors for preeclampsia, 903 women, were included in this cluster analysis. Women who were randomized to receive the low-dose aspirin (n = 69) [13]were excluded. The clinical characteristics of the study groups are presented in Table 2.

**Table 2:** Baseline and pregnancy characteristics.

Characteristics Mean (SD)	Controls with no risk factors for preeclampsia n = 110	One or more risk factors, no preeclampsia n = 817	Preeclampsia n = 86	Early and/or severe preeclampsia n = 36	Late nonsevere preeclampsia n = 50
Age, years	30.0 (4.3)*	32.6 (6.0)*	31.6 (5.4)	32.5 (5.3)	31.0 (5.4)
Weight before pregnancy, kg	62.9 (8.3)*°	76.6 (18.4)*	79.2 (19.7)°	77.6(19.1)	80.4 (20.2)
Weight in the end of pregnancy, kg	77.6 (10.0)*°	89.7(17.9)*	93.1 (19.6)°	91.7 (19.7)	94.0 (19.7)
BMI before pregnancy, kg/m^2^	22.6 (2.7)	27.8 (6.5)	28.6 (6.7)	28.6(7.1)	28.9 (6.8)
BMI at the end of pregnancy, kg/m^2^	27.8 (2.9)	32.5 (6.3)	33.6 (6.9)	33.9 (7.7)	33.8 (6.5)
BMI change, kg/m^2^	5.3 (1.6)	4.6 (2.1)	4.9 (1.8)	4.8(2.1)	4.9(1.9)
Nulliparous	60% *°	31% *	31%(1.0)°	42%	22.%
Blood pressure systolic highest, mmHg	126.2 (13.1)	135.6 (17.0)	169.2 (19.7)	182.3(17.0)^#^	159.9 (15.0)^#^
Blood pressure diastolic highest, mmHg	81.2 (7.8)	88.4 (11.6)	105.0 (10.1)	110.2 (9.8)^#^	101.3 (8.7)^#^
Proteinuria highest, g/24 hour	-	-	2.3 (3.0)	3.6 (4.1)^#^	1.3 (1.2)^#^
Gestational age, weeks	40.1 (1.4)*	39.8 (1.6)^€^	37.6 (3.3)*^€^	35.7 (3.9)^#^	39.0 (1.7)^#^
Birthweight, g	3463 (411)*	3578(550)	3058 (932)*	2420 (921)^#^	3519 (621)^#^
Relative birthweight, SD	-0.2 (0.8)	0.05 (1.1)^€^	-0.5 (1.4)^€^	-1.2 (1.0)^#^	0.1 (1.4)^#^
Placental weight, g	584 (108)*	619 (133)*^€^	560 (170)^€^	464(146)^#^	626 (155)^#^
Uterine artery pulsatility index mean	1.45 (0.38)*	1.51 (0.44)^€^	1.68 (0.47)*^€^	1.74 (0.45)	1.64(0.52)
Mother’s birthweight, g	3513 (488)	3406 (522)	3338 (636)	3410 (598)	3298 (656)

Differences between subjects were calculated by independent-sample t-test for continuous variables and chi-square test for categorical values. BMI = body mass index, relative birthweight = birthweight normalized for gestational age and gender of the newborn. Symbols for statistically significant difference (P<0.05):

* between controls without risk factors and risk women without preeclampsia,

° between controls without risk factors and women with preeclampsia,

^€^ between risk women without preeclampsia and women with preeclampsia,

^#^ between early/severe preeclampsia and late nonsevere preeclampsia,

### Outcomes

Primary outcomes in the study were preeclampsia (blood pressure ≥ 140/90 mmHg in two consecutive measurements and proteinuria ≥ 0.3 g/24hours) [18], gestational hypertension (new onset hypertension after 20 weeks of gestation, without proteinuria), and birth weight standard deviation (SD) score as a continuous variable calculated according to Finnish standards [19].

Secondary outcomes were early-onset preeclampsia (delivery before 34^+0^ weeks of gestation), late-onset preeclampsia (delivery at or after 34^+0^ weeks of gestation), preterm preeclampsia (delivery before 37^+0^ weeks of gestation), term preeclampsia (delivery at or after 37^+0^ weeks of gestation), intermediate preeclampsia (delivery between 34^+0^–36^+6^ weeks of gestation), severe preeclampsia (blood pressure ≥160 mmHg systolic and/or ≥110 mmHg diastolic and/or proteinuria ≥ 5 g/24hours), small for gestational age (SGA) (birthweight <-2SD), gestational diabetes (diet or insulin treated), chronic hypertension (≥140/90 mmHg or medication for hypertension before 20 weeks of gestation), HELLP syndrome (hemolysis, elevated liver enzymes, low platelets) and fetal demise (fetal death after 22^th^ gestational week or over 500g weight).

Each individual outcome diagnosis was set by a jury, which consisted of two physicians and a study nurse. They met face-to-face and reviewed the hospital and maternity clinic records of each participant.

### Methods of the cluster analysis

We applied a Bayesian clustering algorithm based on mixtures of binary variables, see, e.g. [20], using an implementation available in the Bayesian Analysis of Population Structure (BAPS) software [21] to classify the study participants on the basis of their risk factors. The algorithm detected 25 clusters, corresponding to different risk factor combinations. For each cluster detected, we computed the risk ratio of each disease outcome, relative to the risk in the general population. The significance and confidence intervals of the risks in the different clusters were computed using the exact binomial test (function *binom*.*test* in the R software). The false discovery rates (FDR) were computed using function *p*.*adjust* in R.

The risk of preeclampsia and its subtypes in the general Finnish population for these outcomes were estimated according to data from the National Institute for Health and Welfare registers from the year 2013; Medical Birth Register and Care Register for Health Care; preeclampsia 2.5% (from these 24% severe, 8% early-onset, 15% intermediate, 77% term, 23% preterm) with frequences obtained by request from the register authorities and gestational hypertension 4.4%, small for gestational age (SGA) 2.3%, gestational diabetes 9%.

## Results

Of the 903 women 86 (9.5%) developed preeclampsia. Of those with preeclampsia 10 (11.6%) had early-onset disease and 36 (41.9%) severe disease. 465 women (51.5%) did not meet any of the primary or secondary outcome criteria whereas 438 (48.5%) had one or more of these pregnancy complications (Table 3).

**Table 3:** Confirmed diagnoses in the cohort of high-risk women.

Diagnoses	n	%
All preeclampsia	86	9.5
Term preeclampsia	59	6.5
Preterm preeclampsia	27	3.0
Early preeclampsia	10	1.1
Severe preeclampsia	35	3.9
Gestational hypertension	96	10.6
Chronic hypertension	179	19.5
Gestational diabetes, diet-treated	171	18.9
Gestational diabetes, insulin-treated	42	4.7
HELLP syndrome*	6	0.7
Eclampsia	1	0.1
Small for gestational age	34	3.8
Fetal demise	2	0.2
Normal pregnancy	465	51.4

* HELLP = hemolysis, elevated liver enzymes and low platelet count

In the control group of 110 women without risk factors two developed preeclampsia; one of them had severe and the other non-severe late-onset preeclampsia. Eighty-seven percent of women in the healthy control group did not meet any of our primary or secondary outcome criteria.

Systemic lupus erythematosus was inclusion criterion for four women and Sjögren’s syndrome for 13 women. None developed preeclampsia.

Women with preeclampsia had significantly more often induced labour or caesarean sections, and their new-borns had lower Apgar scores (Table 4).

**Table 4:** Delivery characteristics.

	Controls with no risk factors for preeclampsia	High risk women, no preeclampsia	Preeclampsia	p
Induced delivery	13 (11.8%)	230 (28.4%)	57 (67.1%)	<0.0001
Cesarean section	15 (13.7%)	185 (22.7%)	35 (40.7%)	<0.0001
Apgar 1min	8.8 (0.9)*	8.6 (1.3)°	8.0 (1.8)*°	<0.001
Apgar 5 min	9.4 (0.6)*	9.1 (1.0)°	8.5 (1.4)*°	<0.001
Apgar 10 min	9.9 (0.5)*^#^	9.4 (0.8)^#^°	9.0 (0.9)*°	<0.05
Umbilical artery pH	7.2 (0.1)	7.3 (0.1)	7.2 (0.1)	ns

*statistically significant difference between controls with no risk factors and women with pre-eclampsia,

° statistically significant difference between high risk women without preeclampsia and women with pre-eclampsia,

^#^ statistically significant difference between controls with no risk factors and high risk women without preeclampsia tested by Anova with Bonferroni corrections for continuous variables and Pearson’s chi square test for categorical variables

### Results of the cluster analyses

#### Heat map of risk relative to general population

**Fig 1:**
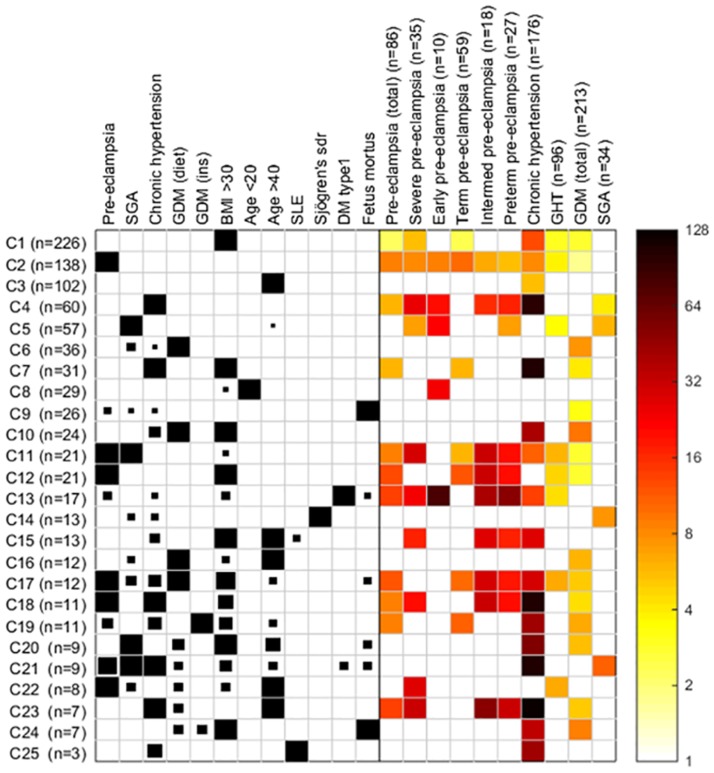
Heat map—Results of the cluster analysis. The heatmap presents the risk factors (columns) in the different clusters on the left side with black boxes. The rows correspond to the 25 clusters (C1-C25) identified on the basis of the risk factor profiles, and the sizes of the clusters are shown on the left side of the heat map (n = 226 etc.). The size (i.e. area) of the black box illustrates the proportion of women in the particular cluster with the risk factor in question. Right side of the heatmap presents the risk ratios of the outcomes. The colour of the cell represents the estimated risk ratio of the corresponding outcome in the corresponding cluster, and the colour encoding is shown on the right side of the heatmap. Those cells are colored which are significant at the nominal 5% level (see text for discussion). The exact risk ratios are presented in Table 5.

The heat map (Fig 1) shows the risk of developing preeclampsia in the high-risk group including severe, early-onset, term, preterm and intermediate preeclampsia as compared to the risk of developing the same outcome in a general population of pregnant women. The risk ratios of the 19 most frequent clusters are presented in Table 5. The risk ratio table including all clusters is presented in the supplement material in S1 Table.

**Table 5:** Risk ratios and 95% Confidence Intervals (CIs) of the cluster analysis. The marking C1 etc. is referring to certain row in the heatmap. If the risk factor is inside brackets, only a portion of women in the cluster had that risk factor.

	End diagnosis, RR (95%CI)					
Risk factors and clusters	Pre-eclampsia (total)	Severe pre-eclampsia	Early pre-eclampsia	Term pre-eclampsia	Intermed pre-eclampsia	Preterm pre-eclampsia
BMI over 30 kg/m (C1)	2.1 (1.1, 3.6)	5.2 (2.1, 10.5)	2.2 (0.1, 12.2)	2.3 (1.1, 4.2)	1.2 (0.02, 6.4)	1.5 (0.2, 5.4)
Pre-eclampsia (C2)	8.1 (5.6, 11.2)	7.2 (2.7, 15.4)	7.2 (0.9, 25.7)	9.2 (6.0, 13.0)	5.7 (1.2, 16.4)	5.0 (1.4, 12.5)
Age over 40 years (C3)	0.4 (0.01, 2.1)	0.0 (0.0, 5.9)	0.0 (0.0, 17.8)	0.5 (0.0, 2.8)	0.0 (0.0, 9.3)	0.0 (0.0, 6.1)
Chronic hypertension (C4)	5.3 (2.4, 9.8)	22.2 (9.9, 41.0)	16.7 (2.0, 57.6)	2.6 (0.5, 7.3)	13.2 (2.7, 36.6)	14.4 (4.8, 31.7)
Small fo gestational age (age >40) (C5)	1.4 (0.2, 4.8)	5.8 (0.7, 20.2)	17.5 (2.1, 60.5)	0.0 (0.0, 3.3)	0.0 (0.0, 16.5)	6.1 (0.7, 20.9)
Gestational diabetes mellitus (sga, ch) (C6)	1.1 (0.03, 5.8)	0.0 (0.0, 16.2)	0.0 (0.0, 48.7)	1.5 (0.04, 7.6)	0.0 (0.0, 25.6)	0.0 (0.0, 16.8)
Chronic hypertension and BMI over 30 (C7)	5.2 (1.5, 11.9)	5.4 (0.1, 27.8)	0.0 (0.0, 56.1)	5.1 (1.1, 13.6)	8.5 (0.2, 44.0)	5.6 (0.1, 28.8)
Age under 20 (bmi) (C8)	2.8 (0.3, 9.1)	5.7 (0.1, 29.6)	17.2 (0.4, 88.8)	1.8 (0.1, 9.4)	0.0 (0.0, 31.4)	5.9 (0.2, 30.6)
Fetus mortus (pe,sga, ch) (C9)	3.1 (0.4, 10.1)	6.4 (0.2, 32.7)	0.0 (0.0, 66.1)	4.0 (0.5, 13.2)	0.0 (0.0, 34.8)	0.0 (0.0, 22.8)
GDM and BMI over 30 (CH) (C10)	0.0 (0.0, 5.7)	0.0 (0.0, 23.7)	0.0 (0.0, 71.2)	0.0 (0.0, 7.5)	0.0 (0.0, 37.5)	0.0 (0.0, 24.6)
Pre-eclampsia and sga (bmi) (C11)	7.6 (2.2, 16.8)	23.8 (5.1, 60.6)	0.0 (0.0, 80.5)	5.0 (0.6, 16.0)	25.1 (3.1, 79.9)	16.4 (2.0, 52.4)
Pre-eclampsia and BMI>30 (C12)	11.4 (4.5, 20.9)	0.0 (0.0, 26.9)	0.0 (0.0, 80.5)	10.0 (2.9, 22.1)	25.1 (3.1, 79.9)	16.4 (2.0, 52.4)
Diabetes mellitus type1 (C13)	11.8 (4.1, 22.4)	19.6 (2.4, 60.7)	58.8 (7.3, 182.2)	3.1 (0.1, 15.1)	31.0 (3.8, 95.9)	40.6 (11.7, 86.0)
Sjögren's syndrome (C14)	0.0 (0.0, 9.9)	0.0 (0.0, 41.2)	0.0 (0.0, 123.5)	0.0 (0.0, 13.0)	0.0 (0.0, 65.0)	0.0 (0.0, 42.6)
BMI and age >40 (C15)	3.1 (0.1, 14.4)	12.8 (0.3, 60.1)	0.0 (0.0, 123.5)	0.0 (0.0, 13.0)	20.2 (0.5, 94.8)	13.3 (0.3, 62.1)
GDM and age >40 (C16)	0.0 (0.0, 10.6)	0.0 (0.0, 44.1)	0.0 (0.0, 132.3)	0.0 (0.0, 13.9)	0.0 (0.0, 69.6)	0.0 (0.0, 45.6)
Pre-eclampsia, GDM(diet), BMI>30 (C17)	10.0 (2.2, 22.9)	0.0 (0.0, 44.1)	0.0 (0.0, 132.3)	8.8 (1.1, 25.5)	21.9 (0.6, 101.3)	14.4 (0.4, 66.3)
Pre-eclampsia, CH, BMI>30 (C18)	7.3 (0.9, 20.7)	15.2 (0.4, 68.8)	0.0 (0.0, 142.5)	4.8 (0.1, 21.7)	23.9 (0.6, 108.6)	15.7 (0.4, 71.2)
GDM insulin treated (pe, ch, bmi)(C19)	7.3 (0.9, 20.7)	0.0 (0.0, 47.5)	0.0 (0.0, 142.5)	9.6 (1.2, 27.3)	0.0 (0.0, 75.0)	0.0 (0.0, 49.1)

Severe pre-eclampsia = blood pressure ≥160 mmHg systolic and/or ≥110 mmHg diastolic and/or proteinuria ≥ 5 g/24hours, early pre-eclampsia = delivery before 34+0 weeks of gestation, term pre-eclampsia = delivery at or after 37+0 weeks of gestation, intermediate pre-eclampsia = delivery between 34+0–36+6 weeks of gestation, preterm pre-eclampsia = delivery before 37+0 weeks of gestation. BMI >30, SGA = small for gestational age (birthweight < 2SD), CH = chronic hypertension, PE = preeclampsia, GDMd = gestational diabetes, diet treated.

The coloured cells in the heatmap signal significant outcomes according to nominal p<0.05 level. After accounting for the number of tests the results correspond to false discovery rate FDR = 0.14 considered appropriate in this exploratory analysis. A similar figure, where significance was determined using the Bonferroni correction over the 25 clusters times 10 outcomes (250 conditions), is shown as Supplementary S1 Fig. Women with preeclampsia in a previous pregnancy, chronic hypertension, a small for gestational age new-born, or type 1 diabetes mellitus were at high risk of early-onset, severe, preterm and intermediate preeclampsia. Preeclampsia in a previous pregnancy and obesity (body mass index (BMI) over 30 kg/m^2)^ were the most important single risk factors for term preeclampsia. Obese women had increased risk of any (OR 2.1, 95%CI 1.1–3.6), term (OR 2.3, 95%CI 1.1–4.2) and severe (5.2, 95%CI 2.1–10.5) preeclampsia. No association with preterm or early-onset preeclampsia was observed. Obesity combined with other risk factors, chronic hypertension, and type 1 diabetes mellitus further increased the risk of preeclampsia. Type 1 diabetes mellitus without other risk factors increased the risk of preterm, but not term preeclampsia. Age over 40 years or under 20 years in a healthy woman without other risk factors did not increase the risk of preeclampsia in our cohort.

**Fig 2:**
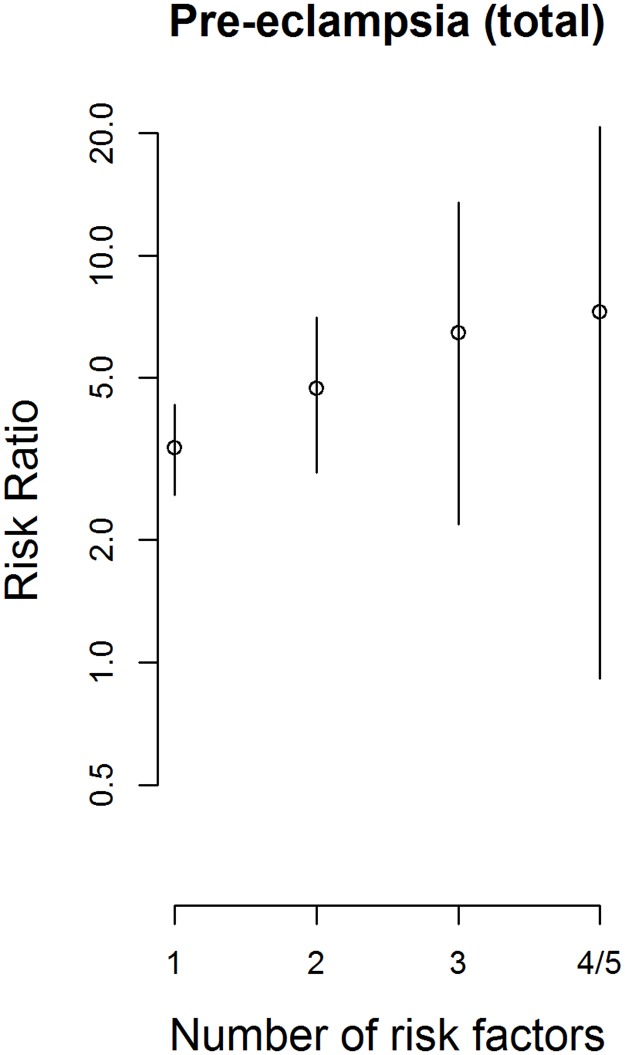
Risk ratio grows exponentially (linearly on the logarithmic scale) as the number of risk factors increases. The increase is significant when the risk of preeclampsia is predicted by the number of risk factors using logistic regression (Preeclampsia (total)(n = 86), p = 0.0181, b = 0.3429)

The risk of preeclampsia increased exponentially (linearly on the logarithmic scale) with increase to the number of risk factors (Fig 2). In Table 6 we present some clusters as an example.

**Table 6:** Important risk factors and clusters and number of women who developed preeclampsia in each group.

Risk factors	Number of women with the risk factor(s)	Number of women who developed preeclampsia (%)
Preeclampsia in a previous pregnancy	138	27 (19.5%)
-Preeclampsia with one or more other risk factors	84	17 (20.2%)
-Preeclampsia and BMI >30kg/m^2^	21	6 (28.6%)
-Preeclampsia and small for gestational age	20	3 (15.5%)
Chronic hypertension	60	8 (13.3%9
-Chronic hypertension with one or more other risk factors	55	13 (24.6%)
-Chronic hypertension and BMI >30kg/m^2^	31	4 (12.9%)
Type 1 diabetes mellitus	11	3 (27.3%)
-Type 1 diabetes mellitus with one or more other risk factors	7	2 (28.8%)
Age over forty years at entry	102	1 (0.9%)
-Age over forty and BMI >30kg/m^2^	19	1 (5.3%)
BMI >30kg/m^2^	227	13 (5.7%)
Sjögren’s syndrome	13	0 (0%)
Systemic lupus erythematosus	4	0 (0%)

## Discussion

In this study the risk of preeclampsia increased exponentially as the number of risk factors increased. Women who developed preterm or severe preeclampsia had a different risk profile than those who developed term preeclampsia. Previous preeclampsia, chronic hypertension, and type 1 diabetes mellitus were strong risk factors for severe and preterm preeclampsia in our cohort. SGA newborn in an earlier pregnancy was a strong risk factor for early-onset preeclampsia. Obesity increased the risk of term preeclampsia and severe preeclampsia. Neither age below 20 years nor age over 40 years, gestational diabetes or fetal demise as a sole risk factors predicted increased risk.

Within this research frame the incidence of preeclampsia in a general population is of particular importance. Incidence of preeclampsia in literature is estimated as 3% [1]. For this work we wanted to determine incidence, which would be accurate in the Finnish population. According to combined data, from the National Medical Birth Registry and Care Register (National Institute for Health and Welfare) 2.5% of babies were born from preeclamptic pregnancies in 2013. Similar rates have been reported in other Nordic countries: according to the Danish hospital discharge registry 2.72% of women who gave birth in Northern Sealand in years 1998–2000 had preeclampsia [22]. In the medical birth registry data of Norway, covering 1 million births in years 1986–2005, preeclampsia rate was 3.7% [23]. While no validation studies exist for preeclampsia diagnoses in the Finnish healthcare registers, studies of the Norwegian [24] and Danish [22] registries have been validated against medical records according to criteria similar to those used in the present study. The prevalence of preeclampsia in the study populations was relatively similar whether obtained from registry or whether based on medical records: 4.4% and 3.8%, respectively, in the Norwegian Study and 2.7% and 2.9% in the Danish study. While some individuals are misclassified according to registry diagnoses, as indicated by the 99% specificity and 69% [22] and 43% [22] sensitivity, the similar prevalences indicate that the registry estimate of prevalence used here as reference is adequately accurate.

The strength of our study is the prospectively recruited, well-characterised cohort of women with increased risk of preeclampsia. However, we had limited information of the onset and severity of preeclampsia in the earlier pregnancies of our participants, and whether preeclampsia had occurred in one or more previous pregnancies. Therefore the effect of those risk factors is impossible to evaluate. According to earlier studies, women with a history of early-onset preeclampsia have higher risk of recurrent preeclampsia than women with late-onset disease [25,26]. Moreover, women with previous preterm preeclampsia have increased risk of adverse pregnancy outcome in their second pregnancy even in the absence of preeclampsia [27].

In this study a history of preeclampsia was a strong risk factor for recurrent preeclampsia. Duckitt and Harrington reported a seven-fold increase in incidence of preeclampsia in women who had preeclampsia in an earlier pregnancy compared to women without such a history [25]. In our cohort, history of giving birth to a SGA new-born in a previous pregnancy seemed to increase the risk of early-onset, preterm and severe preeclampsia in subsequent pregnancies. Conversely, having a preterm preeclampsia in an earlier pregnancy has shown to be associated with risk of giving birth to a SGA newborn in a later non preeclamptic pregnancy [28]. This reflects the common placenta derived pathogenesis of these pregnancy outcomes. Preeclampsia and SGA are different entities of the same placental disease. Ness and Sibai [29] hypothesized that shallow placentation is preceded by endothelial dysfunction in both preeclamptic and SGA pregnancies. If shallow placentation interacts with maternal metabolic disturbances preeclampsia develops, if metabolic disturbances are absent, SGA develops.

Consistent with earlier studies, we found that chronic hypertension is a stronger predictor of early-onset and severe preeclampsia [1]. Chronic hypertension may predispose to placental vascular insufficiency associated with early-onset preeclampsia. Obesity increases the risk of preeclampsia. In our cohort obese women had increased risk of any, term, and severe preeclampsia. No association with preterm preeclampsia was observed. For example in a population based retrospective study with over 854 000 singleton live births the risk ratio for preeclampsia was 2.9 for all women with BMI over 30 kg/m^2^ and the risk increased progressively with the increase in BMI [1]. In our cohort, if a woman had other risk factors together with obesity the risk increased. Obesity together with history of preeclampsia in an earlier pregnancy increased the risk of intermediate and term preeclampsia, but not early-onset preeclampsia.

Women in the extremes of fertile age are thought to be at increased risk of developing preeclampsia [1]. This was not seen in our cohort. Women below 20 years and over 40 years of age did not have increased risk of developing preeclampsia, unless observed together with other risk factors. Women in extremely advanced maternal age, 45 years or over may be in greater risk of preeclampsia spectrum complications [30].

Women with connective tissue diseases, systemic lupus erythematous and Sjögren’s syndrome, are commonly considered at high risk for developing preeclampsia [31]. However, in our cohort none included with these risk factors developed preeclampsia. This may be due to the small sample size. The heterogeneity of these complex diseases may also play a role. It may be that only some subgroups of women, for example those with lupus anticoagulant or renal insufficiency, have an increased risk.

Recent advances in the prevention of preeclampsia in high-risk women have emphasized the identification of these women and the role of individual risk assessment. There is no consensus on the level of risk which would warrant prevention with low dose aspirin and there are only few recommendations concerning the prevention. These recommendations are based on the assumption that low dose aspirin should prevent early-onset and severe preeclampsia more efficiently than late-onset, non-severe disease [12,32]. Society of Obstetric Medicine of Australia and New Zealand [33] recommends low dose aspirin for women at increased risk. The Society of Obstetricians and Gynecologists of Canada [34] recommends low dose aspirin for women at highest risk, women with a history of early-onset preeclampsia and women with recurrent preeclampsia. The recommendations by World Health Organization (WHO) [35], National Institute of Health and Clinical Excellence (NICE) [36], American College of Obstetricians and Gynecologists (ACOG) [2] and U.S. Preventive Services Task Force (USPSTF) [35], provide more detailed guidelines. In July 2016 ACOG released an updated version of recommendations stating that they support the broader list of risk factors associated with high risk by USPSTF[37]. Each of the WHO and NICE and USPSTF recommendations identify previous preeclampsia, diabetes, chronic hypertension, renal disease, and autoimmune disease as factors associated with risk of pre-eclampsia. USPSTF includes also women who have had preeclampsia accompanied by adverse outcome. In the NICE guidelines all hypertensive diseases, including pregnancy hypertension in a previous pregnancy, are evaluated as factors with high risk. In WHO and USPSTF recommendation multiple pregnancy is included as a factor with high risk. The NICE guidelines and USPSTF determine, as well, moderate risk factors and recommends to consider low dose aspirin for women with more than one of these moderate risk factors: first pregnancy, age 35 (in USPSTF) or 40 (in NICE) years or older, pregnancy interval of more than ten years, BMI of 35kg/m^2^ or more, family history of preeclampsia or multiple pregnancy. Accumulation of risk factors increasing the risk of preeclampsia is corroborated by our cluster analysis. For example age over 40 as a sole factor in a healthy participant did not increase the risk of preeclampsia in our cohort, but obese women over forty years of age had an increased risk of preterm preeclampsia. The USPSTF recommendation also includes sociodemographic characteristics in the moderate risk factors. The USPSTF recommendation, unlike others, includes the history of a SGA infant as a moderate risk factor. In our cohort, history of giving birth to a SGA baby in a previous pregnancy seems to increase the risk of early, preterm and severe preeclampsia significantly in subsequent pregnancies.

Gestational diabetes in an earlier pregnancy, obesity or advanced age as sole risk factors does not seem to increase the risk of early-onset or severe preeclampsia. Commercial prediction programs, utilizing placental growth factor, other biomarkers and biophysical measurements are under development. Currently the clinical risk factors are important in estimating the risk of preeclampsia in early pregnancy and consequently the need for preventive low dose aspirin. Based on this cluster analysis we would recommend low dose aspirin for those women who have had preeclampsia or a SGA infant in an earlier pregnancy, women with chronic hypertension and women with type 1 diabetes mellitus if present as a sole risk factor or together with other factors. Clustering of risk factors increases the risk significantly.

We conclude that early-onset and severe preeclampsia has different risk profile from the late-onset preeclampsia. Our analysis indicates exponential increase in the risk of preeclampsia as the number of risk factors increased.

## Supporting information

S1 Table The risk ratios and 95%CI of the least frequent clusters. The marking C20 etc. is referring to certain row in the heatmap. If the risk factor is inside brackets, only a portion of women in the cluster had that risk factor.
(PDF)

S1 Fig The heatmap with Bonferroni corrections. Results of the cluster analysis. The heatmap presents the risk factors (columns) in the different clusters on the left side with black boxes. The rows correspond to the 25 clusters (C1-C25) identified on the basis of the risk factor profiles, and the sizes of the clusters are shown on the left side of the heat map (n = 226 etc.). The size (i.e. area) of the black box illustrates the proportion of women in the particular cluster with the risk factor in question. Right side of the heatmap presents the risk ratios of the outcomes, in this heat map significance is determined using Bonferroni correction over the 25 clusters times 10 outcomes (250 conditions). The colour of the cell represents the estimated risk ratio of the corresponding outcome in the corresponding cluster, and the colour encoding is shown on the right side of the heatmap. Those cells are coloured which are significant at the nominal 5% level.
(TIFF)
