# Supplementary material for: Cluster analysis to estimate the risk of preeclampsia in the high-risk Prediction and Prevention of Preeclampsia and Intrauterine Growth Restriction (PREDO) study
Source: PLoS One. 2017 Mar 28;12(3):e0174399. doi: 10.1371/journal.pone.0174399 (PMC5369775; doi:10.1371/journal.pone.0174399)
Supplement: S1 Table — The marking C20 etc. is referring to certain row in the heatmap. If the risk factor is inside brackets, only a portion of women in the cluster had that risk factor. (PDF) [file pone.0174399.s001.pdf]

Risk factors and clusters	End diagnosis, RR (95%CI) Pre-eclampsia (total)	Severe pre-eclampsia	Early pre-eclampsia	Term pre-eclampsia	Intermed pre-eclampsia	Preterm pre-eclampsia	Chronic hypertension	GHT	GDM (total)	SGA
BMI over 30 kg/m (C1)	2.1 (1.1, 3.6)	5.2 (2.1, 10.5)	2.2 (0.1, 12.2)	2.3 (1.1, 4.2)	1.2 (0.2, 6.4)	1.5 (0.2, 5.4)	11.1 (7.3, 15.9)	2.8 (1.9, 4.0)	2.8 (2.1, 3.4)	1.0 (0.3, 2.2)
Pre-eclampsia (C2)	8.1 (5.6, 11.2)	7.2 (2.7, 15.4)	7.2 (0.9, 25.7)	9.2 (6.0, 13.0)	5.7 (1.2, 16.4)	5.0 (1.4, 12.5)	7.2 (3.5, 12.9)	3.6 (2.3, 5.3)	1.8 (1.1, 2.6)	1.3 (0.3, 3.2)
Age over 40 years (C3)	0.4 (0.0, 2.1)	0.0 (0.0, 5.9)	0.0 (0.0, 17.8)	0.5 (0.0, 2.8)	0.0 (0.0, 9.3)	0.0 (0.0, 6.1)	4.9 (1.6, 11.1)	1.8 (0.8, 3.4)	1.4 (0.8, 2.3)	1.3 (0.3, 3.6)
Chronic hypertension (C4)	5.3 (2.4, 9.8)	22.2 (9.9, 41.0)	16.7 (2.0, 57.6)	2.6 (0.5, 7.3)	13.2 (2.7, 36.6)	14.4 (4.8, 31.7)	80.0 (67.7, 89.2)	0.0 (0.0, 1.4)	1.7 (0.8, 3.0)	3.6 (1.2, 8.0)
Small fo gestational age (age >40) (C5)	1.4 (0.2, 4.8)	5.8 (0.7, 20.2)	17.5 (2.1, 60.5)	0.0 (0.0, 3.3)	0.0 (0.0, 16.5)	6.1 (0.7, 20.9)	3.5 (0.4, 12.1)	3.2 (1.4, 5.9)	0.4 (0.1, 1.3)	5.3 (2.2, 10.3)
Gestational diabetes mellitus (Sga, CH) (C6)	1.1 (0.03, 5.8)	0.0 (0.0, 16.2)	0.0 (0.0, 48.7)	1.5 (0.1, 7.6)	0.0 (0.0, 25.6)	0.0 (0.0, 16.8)	2.8 (0.1, 14.5)	0.0 (0.0, 2.2)	7.1 (5.1, 8.8)	1.2 (0.1, 6.3)
Chronic hypertension and BMI over 30 (C7)	5.2 (1.5, 11.9)	5.4 (0.1, 27.8)	0.0 (0.0, 56.1)	5.1 (1.1, 13.6)	8.5 (0.2, 44.0)	5.6 (0.1, 28.8)	90.3 (74.2, 98.0)	0.7 (0.1, 3.8)	3.9 (2.1, 6.1)	0.0 (0.0, 4.9)
Age under 20 (bmi) (C8)	2.8 (0.3, 9.1)	5.7 (0.1, 29.6)	17.2 (0.4, 88.8)	1.8 (0.1, 9.4)	0.0 (0.0, 31.4)	5.9 (0.2, 30.6)	0.0 (0.0, 11.9)	2.4 (0.5, 6.2)	1.1 (0.2, 3.0)	1.5 (0.0, 7.7)
Fetus mortus (pe, sga, ch) (C9)	3.1 (0.4, 10.1)	6.4 (0.2, 32.7)	0.0 (0.0, 66.1)	4.1 (0.5, 13.2)	0.0 (0.0, 34.8)	0.0 (0.0, 22.8)	3.8 (0.1, 19.6)	0.9 (0.1, 4.5)	3.0 (1.3, 5.3)	0.0 (0.0, 5.8)
GDM and BMI over 30 (CH) (C10)	0.0 (0.0, 5.7)	0.0 (0.0, 23.7)	0.0 (0.0, 71.2)	0.0 (0.0, 7.5)	0.0 (0.0, 37.5)	0.0 (0.0, 24.6)	33.3 (15.6, 55.3)	1.9 (0.2, 6.1)	8.3 (5.9, 10.0)	0.0 (0.0, 6.2)
Pre-eclampsia and sga (bmi) (C11)	7.6 (2.2, 16.8)	23.8 (5.1, 60.6)	0.0 (0.0, 80.5)	5.0 (0.6, 16.0)	25.1 (3.1, 79.9)	16.4 (2.03, 52.4)	9.5 (1.2, 30.4)	5.4 (1.9, 10.7)	2.6 (0.9, 5.2)	2.1 (0.1, 10.4)
Pre-eclampsia and BMI>30 (C12)	11.4 (4.5, 20.9)	0.0 (0.0, 26.9)	0.0 (0.0, 80.5)	10.0 (2.9, 22.1)	25.1 (3.1, 79.9)	16.4 (2.0, 52.4)	4.8 (0.1, 23.8)	4.3 (1.2, 9.5)	2.6 (0.9, 5.2)	0.0 (0.0, 7.0)
Diabetes mellitus type1 (C13)	11.8 (4.1, 22.4)	19.6 (2.4, 60.7)	58.8 (7.3, 182.2)	3.1 (0.1, 15.1)	31.0 (3.8, 95.9)	40.6 (11.7, 86.0)	11.8 (1.5, 36.4)	4.0 (0.9, 9.9)	0.0 (0.0, 2.2)	2.6 (0.1, 12.5)
Sjögren's syndrome (C14)	0.0 (0.0, 9.9)	0.0 (0.0, 41.2)	0.0 (0.0, 123.5)	0.0 (0.0, 13.0)	0.0 (0.0, 65.0)	0.0 (0.0, 42.6)	7.7 (0.2, 36.0)	1.7 (0.04, 8.2)	0.0 (0.0, 2.7)	6.7 (0.8, 19.8)
BMI and age >40 (C15)	3.1 (0.1, 14.4)	12.8 (0.3, 60.1)	0.0 (0.0, 123.5)	0.0 (0.0, 13.0)	20.2 (0.5, 94.8)	13.3 (0.3, 62.1)	23.1 (5.04, 53.8)	3.5 (0.4, 10.3)	2.6 (0.6, 6.0)	3.3 (0.1, 15.7)
GDM and age >40 (C16)	0.0 (0.0, 10.6)	0.0 (0.0, 44.1)	0.0 (0.0, 132.3)	0.0 (0.0, 13.9)	0.0 (0.0, 69.6)	0.0 (0.0, 45.6)	0.0 (0.0, 26.5)	3.8 (0.5, 11.0)	5.6 (2.3, 8.8)	0.0 (0.0, 11.5)
Pre-eclampsia, GDM (diet), BMI>30 (C17)	10.0 (2.2, 22.9)	0.0 (0.0, 44.1)	0.0 (0.0, 132.3)	8.8 (1.1, 25.5)	21.9 (0.6, 101.3)	14.4 (0.4, 66.3)	25.000 (5.5, 57.2)	5.7 (1.2, 13.0)	4.6 (1.7, 8.0)	0.0 (0.0, 11.5)
Pre-eclampsia, CH, BMI>30 (C18)	7.3 (0.9, 20.7)	15.2 (0.4, 68.8)	0.0 (0.0, 142.5)	4.8 (0.1, 21.7)	23.9 (0.6, 108.6)	15.7 (0.4, 71.2)	90.9 (58.7, 99.8)	0.0 (0.0, 6.5)	4.0 (1.2, 7.7)	0.0 (0.0, 12.4)
GDM insulin treated (pe, ch, bmi) (C19)	7.3 (0.9, 20.7)	0.0 (0.0, 47.5)	0.0 (0.0, 142.5)	9.6 (1.2, 27.3)	0.0 (0.0, 75.0)	0.0 (0.0, 49.1)	36.4 (10.9, 69.2)	0.0 (0.0, 6.5)	6.1 (2.6, 9.3)	0.0 (0.0, 12.4)
SGA and BMI (C20) (GDMd, age>40, FM) (C20)	0.0 (0.0, 13.5)	0.0 (0.0, 56.1)	0.0 (0.0, 168.1)	0.0 (0.0, 17.7)	0.0 (0.0, 88.5)	0.0 (0.0, 58.0)	44.4 (13.7, 78.8)	0.0 (0.0, 7.6)	4.9 (1.5, 8.8)	0.0 (0.0, 14.6)
PE, SGA, CH, (GDMd, BMI, AGE>40, T1DM, FM) (	0.0 (0.0, 13.5)	0.0 (0.0, 56.1)	0.0 (0.0, 168.1)	0.0 (0.0, 17.7)	0.0 (0.0, 88.5)	0.0 (0.0, 58.0)	88.9 (51.8, 99.7)	0.0 (0.0, 7.6)	1.2 (0.0, 5.4)	9.7 (1.2, 26.1)
PE and age >40 (SGA, GDMd, BMI) (C22)	5.0 (0.1, 21.1)	20.8 (0.5, 87.8)	0.0 (0.0, 184.7)	6.6 (0.2, 27.7)	0.0 (0.0, 97.2)	0.0 (0.0, 63.7)	0.0 (0.0, 36.9)	5.7 (0.7, 14.8)	1.4 (0.0, 5.9)	0.0 (0.0, 16.1)
CH and age >40 (GDMd) (C23)	11.4 (1.5, 28.4)	23.8 (0.6, 96.5)	0.0 (0.0, 204.8)	7.5 (0.2, 30.5)	37.6 (1.0, 152.3)	24.6 (0.6, 99.8)	100.0 (59.0, 100.0)	0.0 (0.0, 9.3)	4.8 (1.1, 9.1)	6.2 (0.2, 25.2)
BMI and FM (GDMd, GDMi) (C24)	0.0 (0.0, 16.4)	0.0 (0.0, 68.3)	0.0 (0.0, 204.8)	0.0 (0.0, 21.6)	0.0 (0.0, 107.8)	0.0 (0.0, 70.6)	28.6 (3.7, 71.0)	3.2 (0.1, 13.2)	7.9 (3.2, 10.7)	0.0 (0.0, 17.8)
SLE (CH) (C25)	0.0 (0.0, 28.3)	0.0 (0.0, 117.9)	0.0 (0.0, 353.8)	0.0 (0.0, 37.2)	0.0 (0.0, 186.2)	0.0 (0.0, 122.0)	33.3 (0.8, 90.6)	0.0 (0.0, 16.1)	3.7 (0.1, 10.1)	0.0 (0.0, 30.8)

Severe pre-eclampsia = blood pressure ≥160 mmHg systolic and/or ≥110 mmHg diastolic and/or proteinuria ≥ 5 g/24hours, early pre-eclampsia = delivery before 34^{+0} weeks of gestation, term pre-eclampsia = delivery at or after 37+0 weeks of gestation, intermediate pre-eclampsia =delivery between 34+0-36+6 weeks of gestation, preterm pre-eclampsia = delivery before 37+0 weeks of gestation. SGA= small for gestational age (birthweight < -2SD), BMI >30, GDMd =gestational diabetes, diet treated, FM=fetus mortus, PE=preeclampsia, CH=chronic hypertension, T1MD=type 1 diabetes mellitus, GDMi=gestational diabetes, insulin treated, SLE=systemic lupus erythematosus.
