# Supplementary figures and images for: Cluster analysis to estimate the risk of preeclampsia in the high-risk Prediction and Prevention of Preeclampsia and Intrauterine Growth Restriction (PREDO) study
Source: PLoS One. 2017 Mar 28;12(3):e0174399. doi: 10.1371/journal.pone.0174399 (PMC5369775; doi:10.1371/journal.pone.0174399)

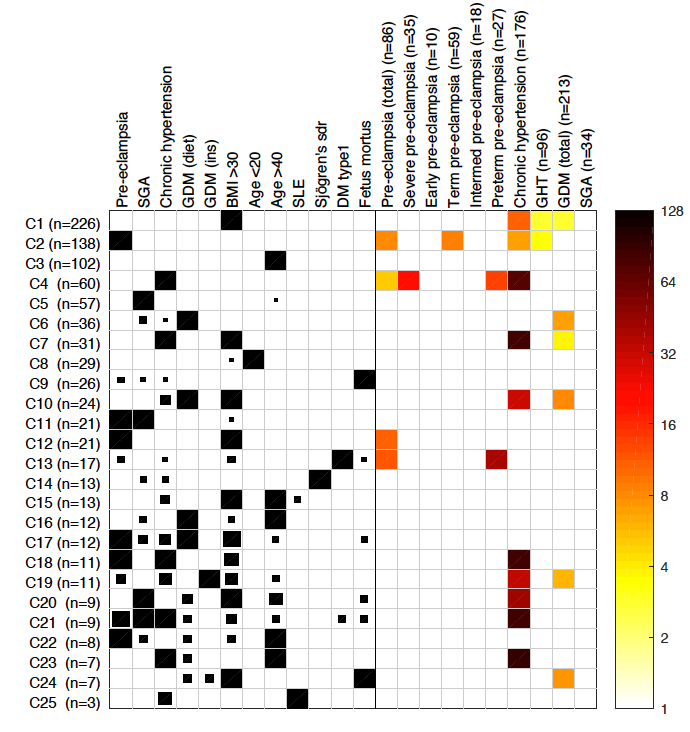

Supplement: S1 Fig — Results of the cluster analysis. The heatmap presents the risk factors (columns) in the different clusters on the left side with black boxes. The rows correspond to the 25 clusters (C1-C25) identified on the basis of the risk factor profiles, and the sizes of the clusters are shown on the left side of the heat map (n = 226 etc.). The size (i.e. area) of the black box illustrates the proportion of women in the particular cluster with the risk factor in question. Right side of the heatmap presents the risk ratios of the outcomes, in this heat map significance is determined using Bonferroni correction over the 25 clusters times 10 outcomes (250 conditions). The colour of the cell represents the estimated risk ratio of the corresponding outcome in the corresponding cluster, and the colour encoding is shown on the right side of the heatmap. Those cells are coloured which are significant at the nominal 5% level. (TIFF) [file pone.0174399.s002.tiff]
